# Age-associated changes in caecal microbiome and their apparent correlations with growth performances of layer pullets

**DOI:** 10.1016/j.aninu.2020.11.019

**Published:** 2021-07-10

**Authors:** Yanli Liu, Tao Yan, Zhouzheng Ren, Xiaojun Yang

**Affiliations:** College of Animal Science and Technology, Northwest A&F University, Yangling 712100, China

**Keywords:** 16S rRNA, Caecal microbiota, Laying hen, Developmental stage, Intestinal health

## Abstract

The microbiome in gastrointestinal tracts play an important role in regulating nutrient utilization and absorption, gut immune function, and host growth or development. This study was conducted to investigate the composition and dynamic distribution of caecal microbiota in pullets during the first 16 weeks. Growth performance, immune organs index, and intestinal morphology of pullets were analyzed at 3, 6, 12 and 16 weeks of age. The caecal contents were collected for microbiota analysis by 16S rRNA gene sequencing method. With advancing ages in pullets, the gradually increased average daily feed intake (ADFI), feed conversion ratio (FCR) and intestinal villus height, but the gradually decreased organs index of thymus and bursa were determined. Meanwhile, more abundant caecal bacterial communities were determined from pullets at 12 and 16 weeks of age than those at 3 and 6 weeks of age. Furthermore, the dominant microflora of pullets from different weeks of age were analyzed by using LEfSe: The higher abundance of *Blautia*, *Prevotella*, *Alistipes,* and *Eggerthella* were found at 6 weeks; *Anaerostipes*, *Oscillospira*, *Enterococcus* and *Methanobrevibacter* were determined at 12 weeks; and the higher abundance of *Parabacteroides*, *Anaerofustis*, *Lactobacillus* and *Butyricimonas* were determined at 16 weeks. Further functional predicted analysis by PICRUSt revealed that the endocrine system and carbohydrate metabolism were significantly developed at 3 weeks. The development of the immune system was predicted to be mainly during 6 to 12 weeks, while cardiovascular diseases and circulatory system were during 12 to 16 weeks. In addition, the significantly negative correlation between *Bacteroides* and villus height, the significantly negative correlation between growth parameters (ADFI and FCR) and *Bacteroides*, *Oscillospira* and *Alistipes*; and the significantly positive relations between growth parameters (ADFI and FCR) and *Bilophila*, *Lactobacillus*, *Rikenella* and *Anaerofustis* were determined by using Pearson analyses. In conclusion, our data demonstrated that growth performance and intestinal morphology correlate well with caecal microbiota, which could provide new insights to establish or develop nutritional strategies to manage the intestinal health or development of laying pullets.

## 1 Introduction

Caecal microbiota and bacterial fermentations, which are crucial to further absorption of nutrients, detoxification of harmful substances, and prevention of pathogen colonization (Yeoman et al., 2012), play important roles in resistance against pathogens, and the regulation of overall health or performance of chickens (Videnska et al., 2014). Numerous studies have shown that gut microbiota could take part in the regulation of feed conversion efficiency (Stanley et al., 2012), glucose metabolism (Mao, 2015), immune function (Woo, 2016), bone formation and growth (Yan et al., 2016) of the host. It is generally accepted that intestinal microbiota composition could be regulated by the physiological, immunological, and nutritional status of the host animals. Many factors have been reported to regulate the intestinal microbiota in chickens, including the developmental stage, genotype, sex, housing environment, and nutritional supplementation (Ding et al., 2017; Kers et al., 2018). One previous study has reported that simultaneous supplementation of AGP and *Bacillus subtilis* DSM17299 for 0 to 3 weeks could increase beneficial microbiota abundance during 0 to 6 weeks and then improve intestinal morphology during 7 to 16 weeks (Li et al., 2018). Neijat et al. (2019) also found that *Bacillus subtilis* DSM29784 could modulate caecal microbiota by selectively enriching the benefical bacteria in grower and developer phases, which in turn promoted the growth and performance of chickens.

Nowadays, the use of antibiotic growth promoter is prohibited, which has prompted research into developing alternative effective probiotics aimed at stimulation of beneficial microbiota in chickens. Furthermore, the cage-free trend also requires alternative ways to ensure health and prevent diseases in egg-laying chickens. Laying hens provide human beings with a main protein source through eggs. A healthy intestinal condition before laying eggs is beneficial and necessary for the whole life of laying hens, which is the precondition of good laying performance during the laying period (Ding et al., 2017). Hence, a better understanding of the composition and dynamic distribution of caecal microbiota along with the growth and development of pullets may provide novel insights into the management of intestinal development and health. Previous studies have also reported that comprehensive analyses of gut microbiota at different developmental stages would contribute to a better understanding of probiotics selection and usage at special physiological stages (Jahromi et al., 2016; Wang et al., 2017; Yan et al., 2017). However, little is known about egg-type birds, in particular during the rearing/development pullet phase. These present situations motivate us to further understand intestinal microbial ecology and its dynamic development in pullets before laying eggs.

The 16S rRNA gene sequencing, which is now widespread in biotechnological applications, has facilitated major advances in our understanding of microbial ecology. In the present study, which aims to characterize the gut microbial establishment of hens before laying eggs and explore whether correlations exist between growth performance and caecal microbiota, 16S rRNA gene sequencing was used to investigate the microbial composition, abundance and dynamic distribution of the caecal microbiota during different developmental stages of laying hens. This study will provide new insights to establish or develop nutritional strategies to manage the intestinal health or development of laying pullets.

## 2 Materials and methods

### 2.1 Experimental design and sample collections

All animal protocols were approved by the Animal Care and Use Committee of the College of Animal Science and Technology of the Northwest A&F University (Shaanxi, China). A total of 90 one-day-old Hy-Line Brown pullets were randomly allotted into 5 replicates of 18 birds each. All birds were kept in an environmentally controlled henhouse with double-layer wired battery cages and had free access to a commercial diet and water at the Experimental Teaching Center of Animal Science in the Norwest A&F University. The room temperature was maintained at 34 to 36 °C for the first week and then decreased by 2 °C per week until reaching 22 to 24 °C. The lighting schedule was 23 h for the first week, and then reduced 2 h per week until reaching 12 h for the duration of the study. During 0 to 3, 4 to 6, 7 to 12 and 13 to 16 weeks of age, average daily feed intake (ADFI), mortality, average daily gain (ADG) and feed conversion ratio (FCR) were calculated. The basal diet was a standard diet commonly used in the northwestern part of China (Li et al., 2018). The composition and nutrient contents of the diet in different phases are included in Appendix Table 1.

At 3, 6, 12 and 16 weeks, one bird of approximately average body weight from every replicate was killed by cervical dislocation and dissected, then the thymus, spleen, and bursa were removed from each bird. The organ weights were immediately measured and were expressed relative to BW (g of organ/kg of BW). Duodenum, jejunum and ileum were excised, and about 3 cm intestinal segments were flushed and fixed in 10% buffered formalin for at least 48 h for histological analysis (Wuhan goodbio technology Co., Ltd). Fresh caecum contents were collected and frozen immediately in liquid nitrogen, and then stored at −80 °C for subsequent analyses.

### 2.2 Microbiota DNA extraction and 16S rRNA sequencing

Microbiota genome DNA were extracted from caecal contents using TIANGEN DNA Kit (TIANGEN, Bening, China) based on the manufacturer's instructions. The 16S rRNA gene was amplified by using the 520F/802R primer set (520F: 5′-AYTGGGYDTAAAGNG-3′, 802R: 5′-TACNVGGGTATCTAATCC-3′), which targets the V4 region of the bacterial 16S rDNA (Pyrobest DNA Polymerase, TaKaRa, Dalian, China). Detailed reaction condition and PCR product purification were based on the previous description (Zhao et al., 2013; Ding et al., 2017). PCR products were purified by using the AxyPrep DNA Gel Extraction Kit (Axygen, Arizona, USA). Pyrosequencing was conducted on an Illumina MiSeq platform and library construction was undertaken using the paired-end sequencing method. Detailed analyses were made in accordance with the previous study (Yang et al., 2017). Trimmed sequences were handled by QIIME for further analysis.

### 2.3 Taxonomy classification

Each sample's trimmed sequence was compared to the Greengene database using the best hit classification option to classify the abundance at the phylum, class, order, family and genus levels in QIIME (http://qiime.org/index.html) (Caporaso et al., 2010). Sequences were clustered to operational taxonomic units (OTU) at an identity threshold of 97% similarity. Alpha-diversity, microbial composition structure and beta-diversity were further analyzed according to the descriptions of a previous study (Ding et al., 2017). LEfSe was applied to identify different taxa microbes among weeks using the default parameters (linear discriminant analysis [LDA] Score >2 and *P* < 0.05). Metastats (http://metastats.cbcb.umd.edu/) was used to calculate taxa abundance at the genus level by pairwise comparison.

### 2.4 Functional predictions analysis

Microbial function analysis was performed using phylogenetic investigation of communities by reconstruction of unobserved states (PICRUSt) (Langille et al., 2013). The OTU were mapped to gallus gallus 13.5 databases at 97% similarity by QIIME's command. The OTU abundance was normalized automatically using 16S rRNA gene copy numbers from known bacterial genomes in Integrated Microbial Genomes (IMG). The predicted genes and their functions were aligned to the Kyoto Encyclopedia of Genes and Genomes (KEGG) database and differences among groups were analyzed by STAMP software.

### 2.5 Statistical analysis

The statistical evaluation of experimental results was analyzed by one-way ANOVA using SPSS 21.0 statistical software with replicates as experimental units except for the 16s rRNA sequencing. All data were expressed as the mean with standard error (SEM). Correlation analysis between growth performance and bacterium were conducted by Pearson correlation procedure of SPSS 21.0. A probability value of *P* < 0.05 was considered to be statistically significant or as a significant correlation. Notable differences among treatments were determined by Duncan's multiple range test. For PICRUSt functional prediction analysis, 2-side Welch's *t*-test and the Benjamini-Hochberg false discovery rate (FDR) (*P* < 0.05) correction were used in groups analysis.

## 3 Results

### 3.1 Growth performance and immune organs index

As shown in Table 1, with advancing ages in pullets, ADFI and FCR were increased gradually (*P* < 0.05). Meanwhile, the ADG during 4 to 6 weeks was higher than the other 3 stages. For immune organs index, the thymus and bursa indexes were decreased gradually with advancing ages in pullets. The highest index of spleen was determined at 6 weeks. There was no mortality through the whole trial.

**Table 1:** Growth performance, immune organs index and intestinal morphology during different stages of pullets for the first 16 weeks.

Item		Weeks				SEM	*P*-value
		3	6	12	16		
Growth performance	ADFI, g/d	17.8^d^	46.4^c^	57.9^b^	83.7^a^	4.57	<0.001
	ADG, g/d	6.8^c^	13.2^a^	10.8^b^	9.9^b^	0.47	<0.001
	FCR	2.6^d^	3.5^c^	5.4^b^	8.6^a^	0.45	<0.001
Organs index^1^	Thymus, g/kg	6.1^a^	4.5^b^	3.5^bc^	2.8^c^	0.30	<0.001
	Spleen, g/kg	1.4^c^	3.6^a^	2.0^b^	1.5^bc^	0.19	<0.001
	Bursa, g/kg	5.4^a^	1.3^b^	0.6^c^	0.5^c^	0.39	<0.001
Duodenum	Villus height, μm	433.6^b^	616.3^a^	610.3^a^	622.5^a^	18.6	<0.001
	Crypt depth, μm	55.2^b^	70.5^a^	48.0^c^	50.1^bc^	2.01	<0.001
	V/C	7.9^b^	8.8^a^	13.0^c^	12.4^bc^	0.52	<0.001
Jejunum	Villus height, μm	235.5^b^	297.8^b^	371.3^a^	416.9^a^	16.7	<0.001
	Crypt depth, μm	41.8^b^	49.4^a^	51.4^a^	51.0^a^	1.21	0.007
	V/C	5.7^c^	6.1^bc^	7.2^ab^	8.2^a^	0.28	0.003
Ileum	Villus height, μm	213.9^c^	281.4^b^	362.3^a^	349.9^a^	14.7	<0.001
	Crypt depth, μm	48.1	44.5	43.6	48.4	0.94	0.152
	V/C	4.5^d^	6.3^c^	8.3^a^	7.2^b^	0.32	<0.001

ADFI = average daily feed intake; ADG = average daily gain; FCR = feed conversion rate; V/C = the ratio of villus height to crypt depth.

^a-d^ Means within a row with different letters are significantly different (*P* < 0.05).

1 Organs index = Organ weight (g)/Body weight (kg).

### 3.2 Intestinal morphology

As exhibited in Table 1, the highest duodenal villus height was determined in pullets at 6 weeks of age and tended to be stable later. The jejunal and ileal villus height both plateaued at 12 weeks of the age. The maximum crypt depth of the duodenum and jejunum were determined in pullets at 6 weeks of age, however, the crypt depth of the duodenum declined during 12 to 16 weeks but the crypt depth in the jejunum maintained a steady state during 6 to 16 weeks. The highest duodenal ratio of villus height to crypt depth was determined in pullets at 6 weeks of age, and then decreased at 12 and 16 weeks of age. In the jejunum, the ratio increased gradually in pullets from 3 to 16 weeks of age. In the ileum, it increased gradually among 3, 6 and 12 weeks and then decreased at 16 weeks.

### 3.3 Richness and diversity of bacteria

**Fig. 1:**
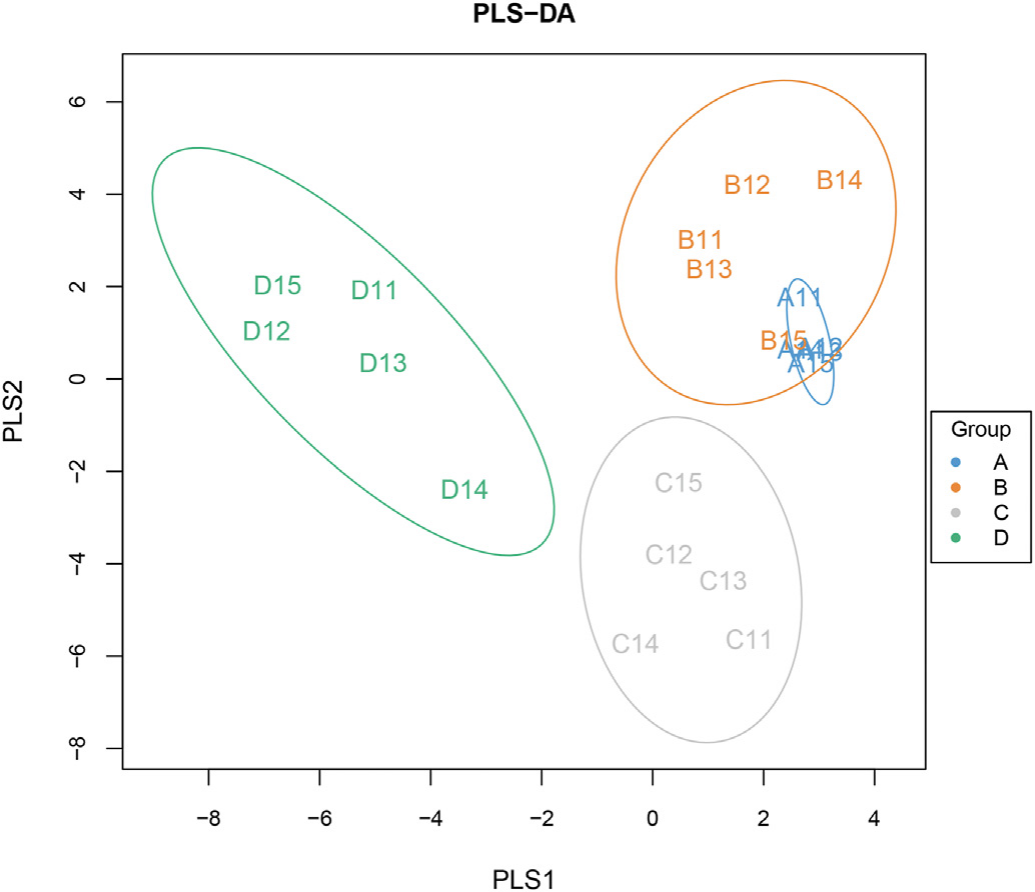
Partial least squares discriminant analysis (PLS-DA) of chicken caecal microbiota among 4 developmental stages of pullets for the first 16 weeks (*n* = 5). A, B, C and D represent the developmental periods of 3, 6, 12 and 16 weeks, respectively.

As shown in Appendix Fig. 1, a total of 563 OTU were shared in the pullets of 3, 6, 12, and 16 weeks of age, and 6, 16, 28 and 47 unique OTU were respectively determined at these 4 different developmental stages of pullets. Alpha-diversity indexes (Chao1, ACE, Shannon and Simpson) were investigated and are shown in Table 2. Higher species richness values (Chao1 and ACE indexes) were determined in pullets at 12 and 16 weeks of age than those at 3 and 6 weeks of age (*P* < 0.05), and the bacterial diversity (Shannon and Simpson indexes) exhibited a similar phenomenon. These results indicated that the microbial diversity and abundance were all increased along with the growth and development of pullets. In addition, a partial least squares discriminant analysis (PLS-DA) showed significant differences of microbial communities among different weeks of pullets (Fig. 1).

**Table 2:** The richness and diversity of caecal microbiome during different stages of pullets for the first 16 weeks.

Item	Weeks				SEM	*P*-value
	3	6	12	16		
Shannon	5.73^b^	5.84^b^	6.53^a^	6.45^a^	0.121	0.020
Simpson	0.94^b^	0.94^b^	0.97^a^	0.96^a^	0.004	0.037
Chao1	526.19^b^	494.90^b^	608.82^a^	604.67^a^	12.765	0.035
ACE	526.30^b^	498.36^b^	616.06^a^	608.47^a^	11.492	0.038

^a, b^ Means within a row with different letters are significantly different (*P* < 0.05).

### 3.4 Microbiota composition at different developmental periods

**Fig. 2:**
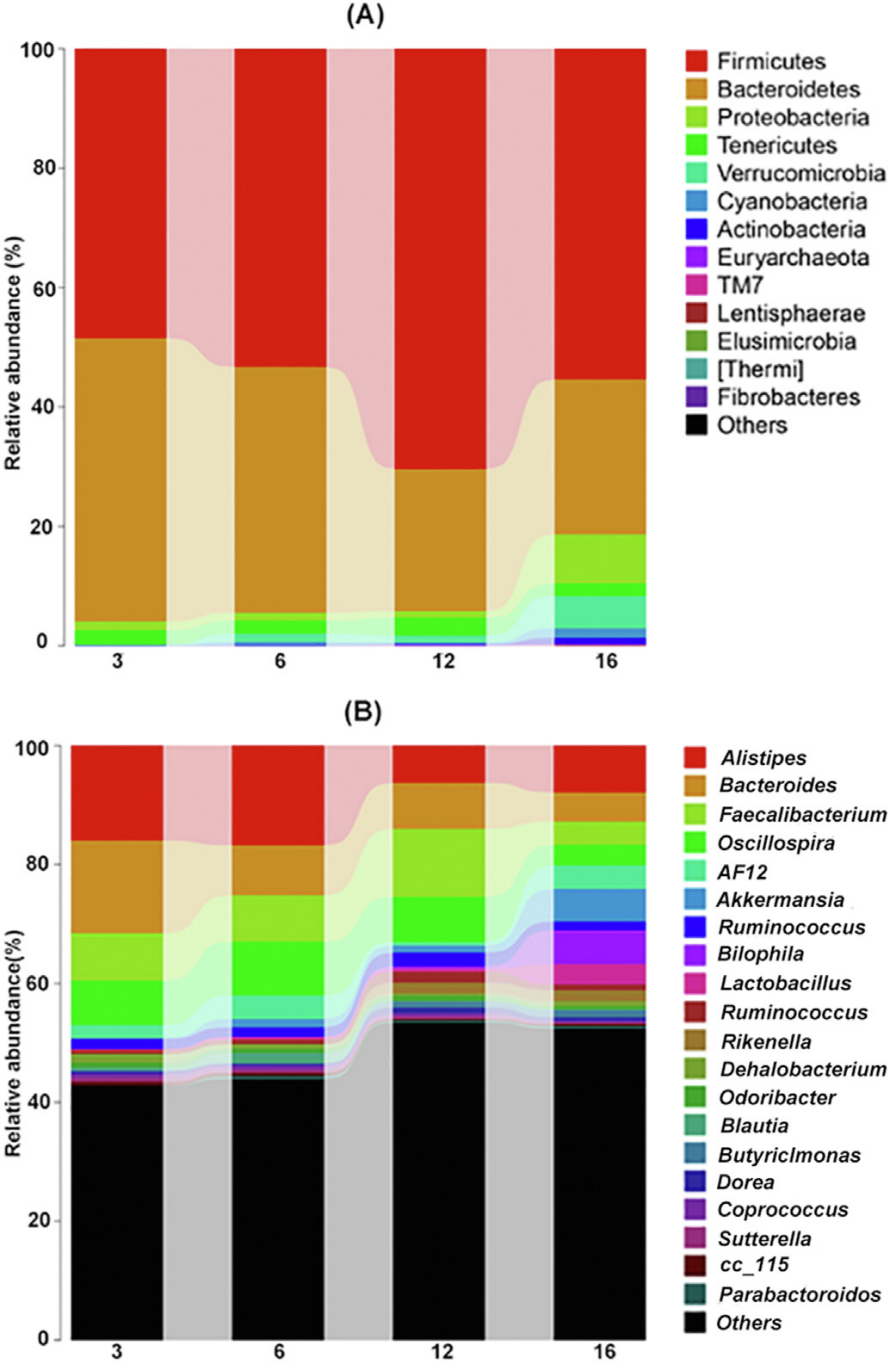
Relative abundance of bacterial composition in caecal contents at phyla **(A)** and genus **(B)** levels among different stages of pullets among 3, 6, 12 and 16 weeks. Each color represents one bacteria. The X-axis shows the week of the age for pullets and the Y-axis shows the percentage of the bacteria.

In order to investigate the caecal microbiota composition of pullets at different developmental periods before laying eggs, we compared the composition and abundance of caecal microbiota at 3, 6, 12 and 16 weeks of age. As shown in Fig. 2A, the most abundant phyla in all periods were Firmicutes and Bacteroidetes. However, the proportions of these 2 phyla among 4 periods were different. The percentage of Firmicutes was increased and the percentage of Bacteroidetes was decreased at the first 12 weeks, and then maintained stability in the period of 12 to 16 weeks. As the thirdly abundant phyla, Proteobacteria significantly increased to 8% among total caecal microbes in 16-week-old pullets, holding at only 2% during the other 3 periods.

As presented in Fig. 2B, at the genus level, *Alistipes*, *Bacteroides*, *Faecalibacterium* and *Oscillospira* were the major bacterial genera in pullets at 3, 6 and 12 weeks of age. Among these, the proportion of *Alistipes* significantly decreased in the 12-week-old and 16-week-old pullets when compared with the pullets at 3 and 6 weeks of age. However, the proportions of *Bilophila* and *Lactobacillus* were significantly increased in pullets at 16 weeks of age when compared with those at the other 3 stages.

**Fig. 3:**
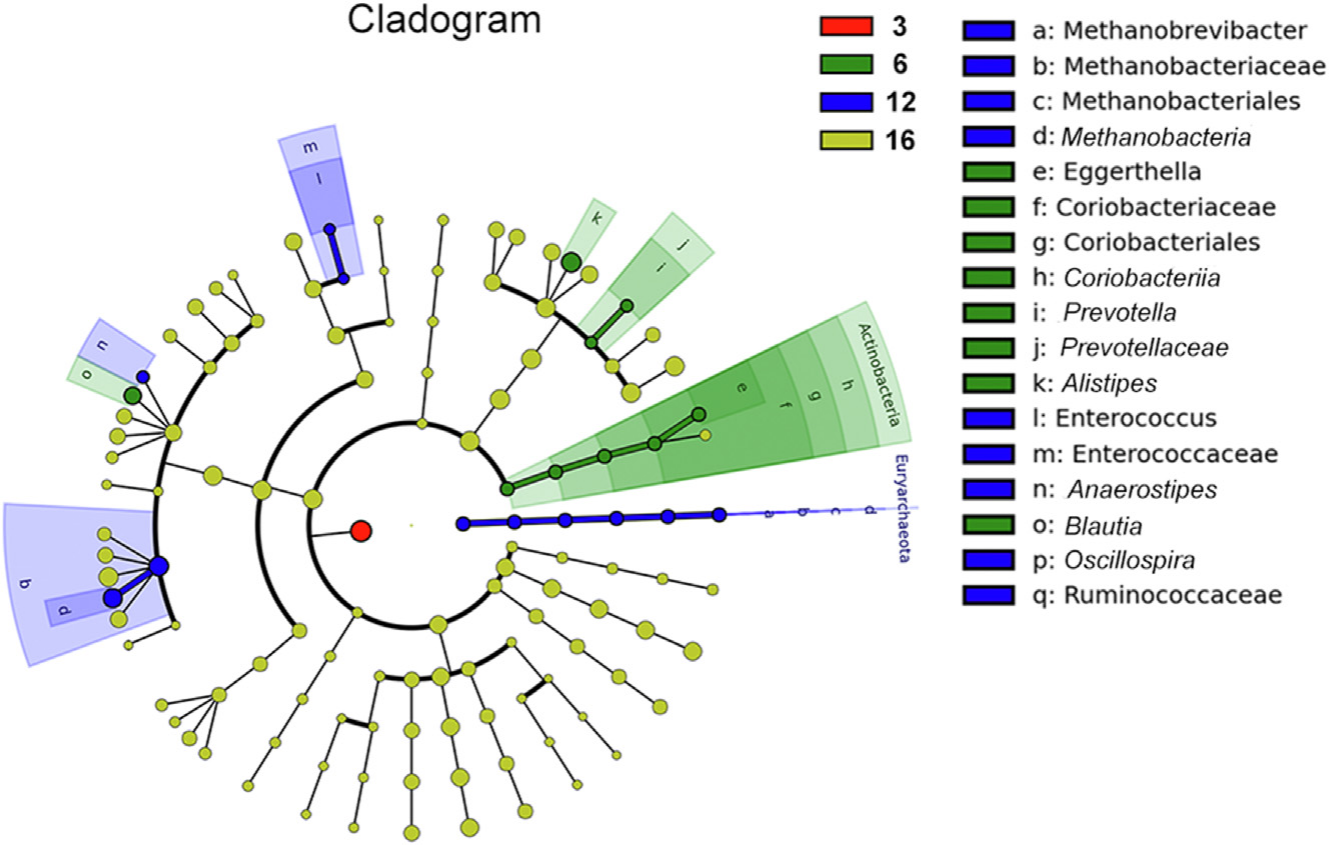
Taxonomic cladogram of chicken caecum microbes at 3, 6, 12 and 16 weeks. The colored circles from inside to out represent the classification level (phylum, class, order, family and genus). The color of circles with letters mean that the bacteria was higher at specific weeks of the age. 3, 6, 12 or 16 weeks are colored by red, green, blue or yellow, respectively.

**Fig. 4:**
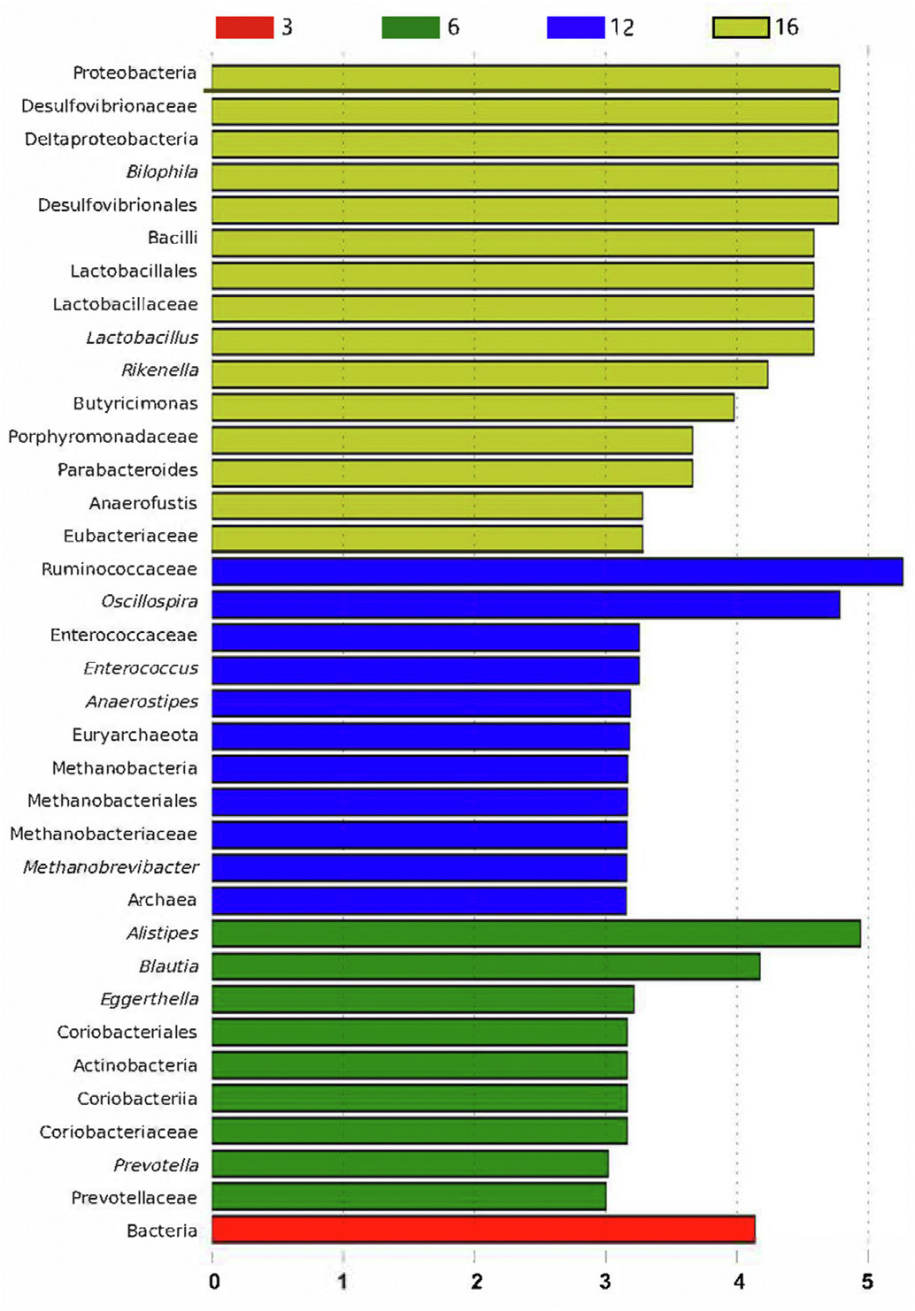
Different taxa microbes analysis in caecum based on LEfSe method at 3, 6, 12 and 16 weeks of chicken. The default parameters were LDA score >2 and *P* < 0.05. Bacterium with red, green, blue or yellow colors mean that they are higher at 3, 6, 12 and 16 weeks respectively.

**Fig. 5:**
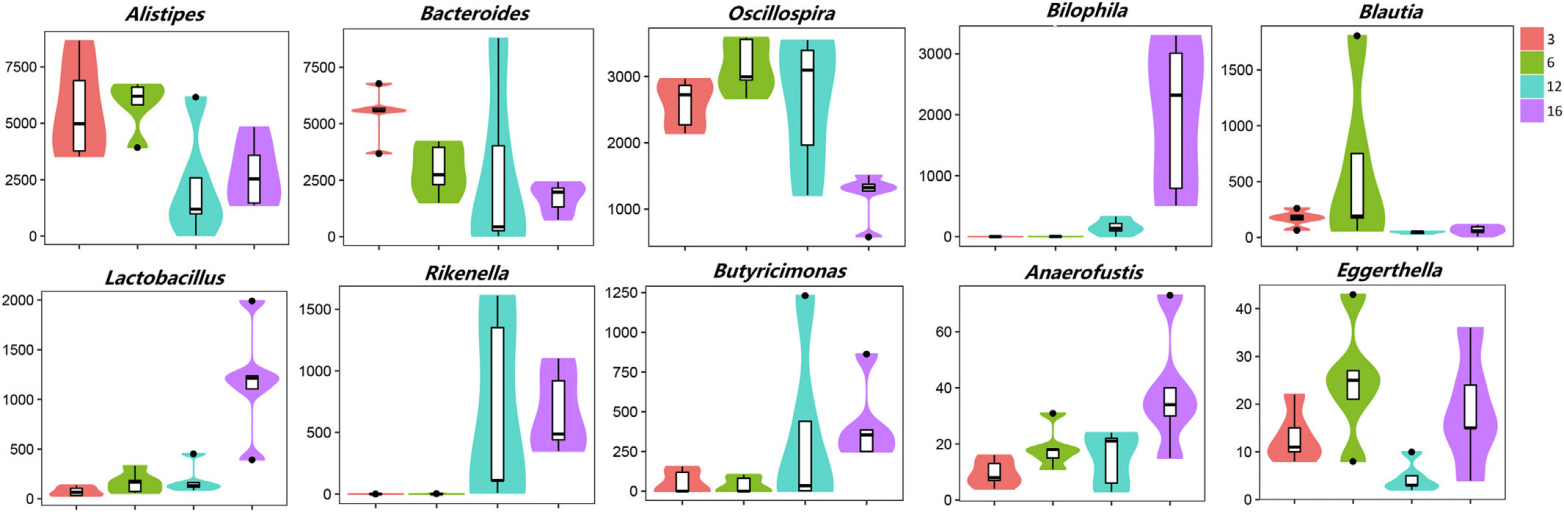
The dynamic distribution of first 10 significant microbes within different development stages of pullets for the first 16 weeks.

According to the cladogram of the microbiota structure axis, a significant shift of microbiota was determined in pullets at 3, 6, 12 and 16 weeks of age (Fig. 3). From LEfSe analysis in Fig. 4, the higher abundance of *Blautia, Prevotella, Alistipes, and Eggerthella* were found at 6 weeks*;* the higher abundance of *Anaerostipes, Oscillospira, Enterococcus,* and *Methanobrevibacter* were determined at 12 weeks; and the higher abundance of *Parabacteroides, Anaerofustis, Lactobacillus, and Butyricimonas* were determined at 16 weeks*.* Based on the Metastats analysis, the abundance of the 10 most significantly altered genera are exhibited in Fig. 5, and *P* values of pairwise comparisons among the 4 developmental periods are further summarized in Appendix Table 2.

### 3.5 Functional predicted analysis of caecal microbiome at different developmental periods

**Fig. 6:**
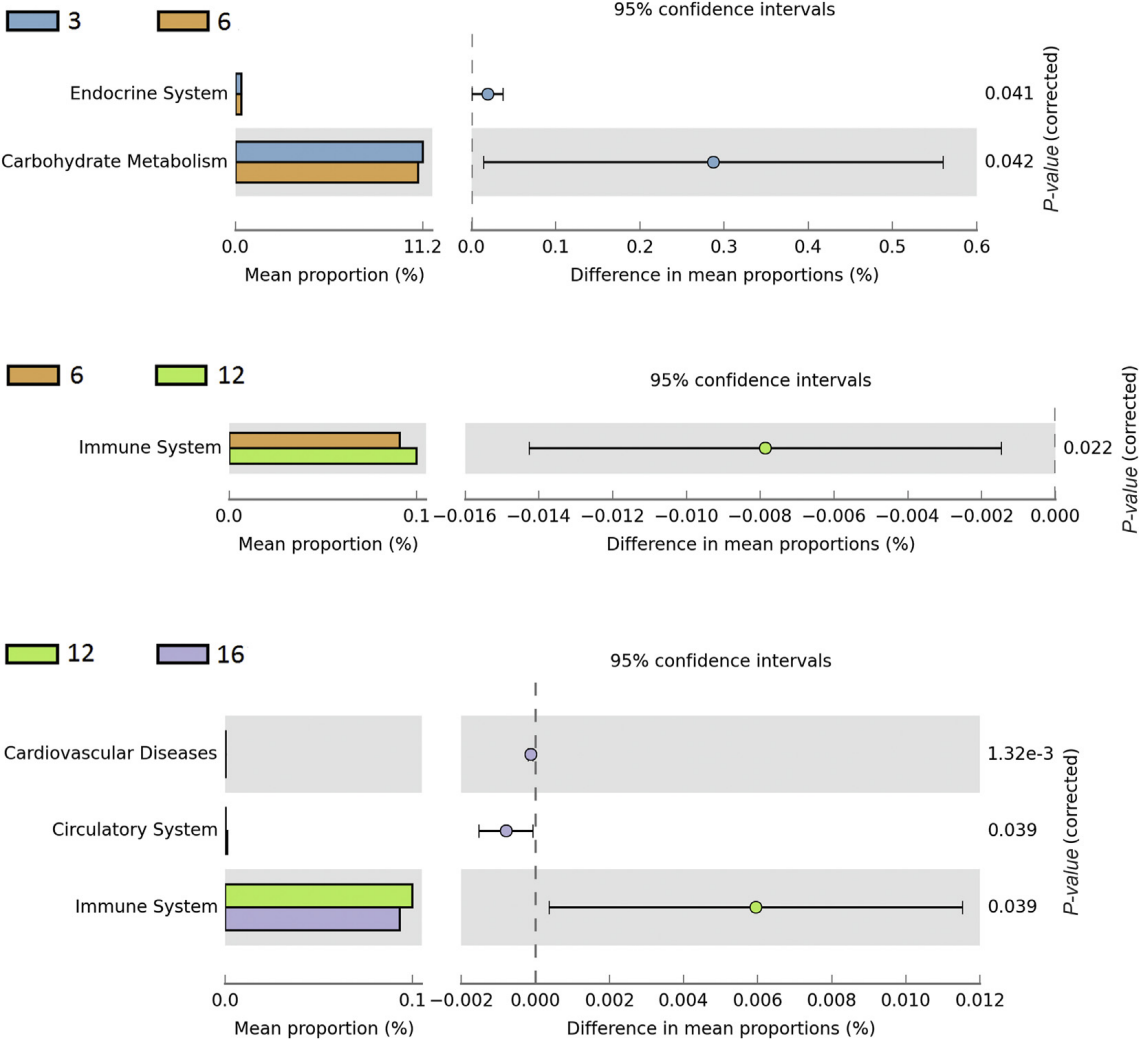
Comparisons of functional pathways between microbes during different stages (3 vs. 6, 6 vs. 12, 12 vs. 16 weeks) of pullets for the first 16 weeks.

The functional difference of the caecal microbiome between 2 adjacently developmental stages was analyzed by using PICRUSt. As shown in Fig. 6, the microbiota of pullets at 3 weeks of age have significantly enriched functional capacities involved in the endocrine system and carbohydrate metabolism when compared with the microbiota of pullets at 6 weeks of age. The significantly enriched functional capacities related to the immune system were predicted to hold a dominant position at 12 weeks, while cardiovascular diseases and the circulatory system were significantly improved at 16 weeks. These functional differences of caecal microbiota in pullets among different developmental stages suggested a correlation between the growth development of pullets and the microbial functional capacity.

### 3.6 Correlation analysis between growth performance and bacterium

In order to reveal the internal relationships between the caecal microbiome and the growth development of pullets, further Pearson correlation analysis was performed. As shown in Table 3, a significantly negative correlation between *Bacteroides* and villus height was determined. Meanwhile, the thymus index was positively correlated with the abundance of *Alistipes*, *Bacteroides*, *Dehalobacterium* and *Oscillospira*, and was negatively correlated with the abundance of *Bilophila*, *Lactobacillus*, *Rikenella* and *Anaerofustis* in the caecum. In addition, the significantly negative correlation between growth parameters (ADFI and FCR) and *Bacteroides*, *Oscillospira*, and *Alistipes*, and the significantly positive relations between growth parameters (ADFI and FCR) and *Bilophila*, *Lactobacillus*, *Rikenella* and *Anaerofustis* were also determined.

**Table 3:** Correlation analysis between growth performance and bacterium in caecum during different stages of pullets for the first 16 weeks.

Item		G1	G2	G3	G4	G5	G6	G7	G8	G9	G10	G11	G12
Duodenum	Villus height	/	#	/	/	/	/	/	/	/	/	/	/
	Crypt depth	∗	/	/	/	/	/	##	/	∗	/	/	/
	V/C	#	/	/	/	/	/	∗∗	/	/	/	/	/
Jejunum	Villus height	#	#	/	/	∗	/	/	#	/	/	/	/
	Crypt depth	/	/	/	/	/	/	/	/	/	/	/	/
	V/C	/	/	/	/	∗	/	/	#	/	/	/	/
Ileum	Villus height	##	##	/	/	/	/	∗	/	/	/	/	/
	Crypt depth	/	/	/	/	/	/	/	/	/	∗	/	/
	V/C	##	#	/	/	/	/	∗	#	/	/	∗	/
Organ index	Thymus	∗	∗	∗	##	##	/	##	∗	/	#	/	/
	Spleen	/	/	∗	/	/	/	/	/	∗∗	/	/	/
	Bursa	/	∗	/	/	/	/	#	∗∗	/	/	/	/
Performance	ADFI	#	##	##	∗∗	∗∗	/	∗	/	/	∗∗	/	/
	ADG	/	/	/	/	/	/	/	#	/	/	/	/
	FCR	#	#	##	∗∗	∗∗	/	∗∗	/	/	∗∗	/	/

G1 = Alistipes; G2 = Bacteroides; G3 = Oscillospira; G4 = Bilophila; G5 = Lactobacillus; G6 = Ruminococcus; G7 = Rikenella; G8 = Dehalobacterium; G9 = Blautia; G10 = Anaerofustis; G11 = Anaerotruncus; G12 = Eggerthella; V/C = the ratio of villus height to crypt depth; ADFI = average daily feed intake; ADG = average daily gain; FCR = feed conversion rate.

“/” means that there is no significant correlation (*P* > 0.05); “∗” or “∗∗” means that there is a significant positive correlation between 2 indices at the 0.05 or 0.01 level (2- tailed); “# “or “## ”means that there is a significant negative correlation between 2 indices at the 0.05 or 0.01 level (2- tailed).

## 4 Discussion

The microbiome in gastrointestinal tracts plays an important role in regulating the intestinal utilization and absorption of nutrients, gut immune function, and the growth and development of their host (Yeoman et al., 2012). Its composition reflects the co-evolution relationship among the inhabiting microbes, the genetic, immune and metabolic condition of the host, and environmental factors (Yeoman et al., 2011). With increasing pressures related to the ban on antibiotic growth promoters in animals, the composition and function of the chicken microbiome has received more attention. So far, the main focus of microbiota research in chickens has been to understand how the microbiota was affected by different feeding strategies and how the microbiota further influenced the performance of hosts (Liu et al., 2017; Baldwin et al., 2018). However, a limited body of research has focused on the recruitment and establishment of the caecal microbiome in pullets during different developmental stages before laying eggs, which could provide more information about implementing viable nutritional strategies to manage the intestinal health and growth development of pullets.

A previous study has reported that nearly 6 to 7 weeks are needed to established the caecal microbial community from the time that chickens are born (Coloe et al., 1984). Following this, the microbiome will continually change and diversify along with the development and growth of chickens (Lu et al., 2003). In the present study, our data showed that the richness and diversity of the bacterial community in pullets at 12 weeks of age was higher than at earlier time points, which suggested that the establishment of caecal microbiota in pullets was not complete before 12 weeks. Meanwhile, Firmicutes and Bacteroidetes were the 2 most abundant phyla in the caecum of pullets during all 16 weeks, which was consistent with a previous study (Waite and Taylor, 2014). Proteobacteria reached the third dominating phyla at 16 weeks, which was reported as one of the 3 most abundant phyla in broilers at 3 and 6 weeks of age (Ding et al., 2017). These differences could be attributed to the different species (laying hens versus broilers) used in the present study and previous studies. Neijat et al. (2019) also found that Proteobacteria represented a significant proportion in grower (5 to 10 weeks), and developer (11 to 16 weeks) phases of pullets.

The microflora in the caecum can metabolize undigested nutrients into end products, which have positive roles in stimulating gut development, immune system development, and nutrients absorption (Adil and Magray, 2012). Different microorganism have their own special metabolism functions, which indicates that gut microbiota could co-evolve with their host animals and further influence different physiological functions of the host animals. *Bacteroides, Blautia*, *Prevotella*, *Alistipes* and *Eggerthella* were the dominant bacteria in pullets at 6 weeks of age. Of these, *Blautia*, *Prevotella* and *Alistipes* can express enzymes involved in the synthesis of propionate (Polansky et al., 2016), which is an important short-chain fatty acid (SCFA) and can take part in the gut development as an important nutrient source. Meanwhile, it was reported that *Blautia* genus was associated with a reduction in death from graft-versus-host disease (Jenq et al., 2015). *Bacteroides* are associated with the degradation of the isoflavone genistein (Renouf and Hendrich, 2011). Here, the isoflavone genistein could ameliorate metabolic and immunological dysfunction in high-fat-diet induced obese mice (Gauffin et al., 2012), which indicated that a high abundance of *Bacteroides* is beneficial for gut health. In addition, *Alistipes* was characterized by high expression of xylose isomerase and glutamate decarboxylase, which could further metabolize glutamate into another important SCFA named γ-aminobutyric acid (Polansky et al., 2016). Intestinal *Prevotella* abundance was strongly associated with long-term supplementation of diets enriched with carbohydrates (Wu et al., 2011), indicating that *Prevotella* could utilize carbohydrates and improve the digestibility of carbohydrates. Overall, these dominant bacteria were all beneficial for the development and growth of host pullets. Moreover, carbohydrate metabolism was also significantly enriched in pullets at 3 and 6 weeks of age, suggesting that carbohydrate metabolism is vigorous during the first 6 weeks. For laying pullets, the first 6 weeks is a critical period for the development of immune and digestive organs, indicating that carbohydrates are also crucial for the hosts’ growth during the first 6 weeks. Meanwhile, feed efficiency in the first 6 weeks was continually increased, and bacterial genus of *Alistipe and Bacteroides* were all positively correlated with increased ADFI and FCR, which was consistent with a previous publication that *Alistipes* was found to have strong relationship with nutrient retention variables associated with growth performance in 5- to 10-week-old pullets (Neijat et al., 2019). Taken together, it was speculated that these bacteria may be related to carbohydrate metabolism and could be used as alternative probiotics for pullets at the first 6 weeks.

*Anaerostipes*, *Oscillospira*, *Enterococcus* and *Methanobrevibacter* were determined as higher abundant genera at 12 weeks when compared with the other 3 developmental stages. *Anaerostipes* was reported to express small acid-soluble spore protein and spore coat protein, which occupy more than 40% preference for carbohydrate and energy metabolism respectively (Polansky et al., 2016). *Oscillospira* can produce secondary bile acids by degrading glycans in the gut, and protect against infection with *Clostridium difficile* (Konikoff and Gophna, 2016)*. Enterococcus*, which appears as a natural colonizer of the intestine in most humans and animals, was used as a probiotic in the previous study (Mountzouris et al., 2007; Arias and Murray, 2012). Moreover, *Enterococcus* was known to cause a range of infections, but generally showed low virulence. To a lesser extent, the bacterial genus with low toxicity could promote the development of immune system of hosts, and further resist exogenous pathogens, which indicated that the *Enterococcus* may be beneficial in promoting the immune function. In the current study, function prediction analysis also showed that the immune system attained its utmost developmental stage at 12 weeks of age when compared with its 2 adjacent phases (6 and 16 weeks of age), suggesting that the developmental period of the immune function was mainly during 6 to 12 weeks in pullets.

During 12 to 16 weeks, circulatory system and cardiovascular diseases were predicted based on function analysis by using PICRUSt. *Lactobacillus*, the preponderant caecal strains at 16 weeks in the present study, showed a tendency to promote the proliferation of the beneficial taxa but reduce the proliferation of the pathogenic taxa, and further improved the body weight of broilers in the previous study (Baldwin et al., 2018). *Butyricimona*, as the dominant bacteria in this stage, produced butyrate by expressing acetyl-CoA acetyl-transferase (Polansky et al., 2016). Previous studies reported that butyrate could serve as a source of energy for colonic epithelial cells (Fleming et al., 1991), and suppress the expression of virulence factors of bacterial pathogens (Boyen et al., 2008). *Parabacteroides* was reported to have anti-inflammatory and anti-cancer properties by the suppression of Toll-like receptor 4 (*TLR*4) and protein kinase B (*Akt*) signaling and the promotion of apoptosis process (Koh et al., 2018), indicating that *Parabacteroides* might be related to diseases defense. Overall, *Lactobacillus* and other dominant bacterium might play vital roles for the special physiological developmental need of pullets in this phase when pullets are preparing for egg production.

## 5 Conclusion

In summary, based on caecal microbiota analysis in the current study. Firmicutes and Bacteroidetes formed the vast majority of microbiota across the first 16 weeks in laying pullets. Growth performance and intestinal morphology were found to be related to caecal microbiota. Potential function prediction analysis indicated that caecal microbiota might take part in the regulation of metabolism and development of pullets in different growth phases, which could provide an excellent platform for leveraging these findings into probiotics additives. To a lesser extent, *Alistipes* and *Prevotella* might be suitable probiotic candidates for layers during the first 6 weeks, while *Lactobacillus* may be more suitable for 6 to 12 and 12 to 16 weeks.

## Author contributions

**Yanli Liu:** Conceptualization; Methodology; Data curation; Writing-Original draft preparation. **Tao Yan**: Sample collection; Software. **Zhouzheng Ren**: Visualization; Investigation; Writing-review and editing. **Xiaojun Yang**: Supervision; Writing-reviewing and editing; Project administration; Funding acquisition.

## Conflict of interest

We declare that we have no financial and personal relationships with other people or organizations that can inappropriately influence our work, and there is no professional or other personal interest of any nature or kind in any product, service and/or company that could be construed as influencing the content of this paper.
