# Supplementary material for: Age-associated changes in caecal microbiome and their apparent correlations with growth performances of layer pullets
Source: Anim Nutr. 2021 Jul 10;7(3):841–8. doi: 10.1016/j.aninu.2020.11.019 (PMC8379648; doi:10.1016/j.aninu.2020.11.019)
Supplement: Multimedia component 1 [file mmc1.docx]

**Supplementary Tables**

**Table S1** Composition and nutrient levels of basal diets (as-fed basis)

| Items | 0-3 weeks | 4-6 weeks | 7-12 weeks | 13-16 weeks |
| --- | --- | --- | --- | --- |
| **Ingredients (%)** | | | | |
| Corn | 42.91 | 61.00 | 63.67 | 63.97 |
| Soybean meal | 17.00 | 23.00 | 22.00 | 18.00 |
| Extruded corn | 20.00 | - | - | - |
| Fermented soybean meal | 6.00 | 4.00 | - | - |
| Fish meal | 3.00 | 2.00 | 1.00 | - |
| Wheat bran | 3.00 | 5.00 | 10.00 | 15.00 |
| Chicken plasma gluten meal | 2.00 | - | - | - |
| Soybean oil | 1.80 | 1.40 | 0.05 | - |
| Glucose | 1.50 | - | 0.30 | - |
| CaHPO_4_ | 1.20 | 1.40 | - | - |
| Limestone | 1.00 | 1.20 | 1.30 | 1.30 |
| NaCl | 0.12 | 0.20 | 1.20 | 1.20 |
| Lys | 0.10 | 0.10 | 0.30 | 0.34 |
| Choline chloride | 0.10 | 0.06 | - | - |
| *DL*-Met | 0.05 | 0.06 | - | - |
| [Acidifier](E:/Dict/6.3.69.8341/resultui/frame/javascript:void(0);) | 0.04 | 0.40 | 0.02 | 0.03 |
| Complex enzyme | 0.03 | 0.03 | - | - |
| Thr | 0.02 | 0.02 | 0.03 | 0.03 |
| Multi-minerals^1^ | 0.10 | 0.10 | - | - |
| Multi-vitamin^2^ | 0.03 | 0.03 | 0.10 | 0.10 |
| Total | 100.00 | 100.00 | 0.03 | 0.03 |
| **Nutrient levels (%)** | | | | |
| CP | 20.00 | 19.70 | 17.50 | 16.00 |
| Ca | 0.82 | 0.91 | 0.83 | 0.79 |
| Total P | 0.62 | 0.63 | 0.61 | 0.61 |
| Available phosphorus | 0.38 | 0.40 | 0.36 | 0.35 |
| NaCl | 0.37 | 0.34 | 0.37 | 0.37 |
| Lys | 1.18 | 1.11 | 0.87 | 0.75 |
| Met | 0.39 | 0.39 | 0.31 | 0.29 |
| Met+Cys | 0.75 | 0.72 | 0.63 | 0.59 |

^1^The trace element premix provided per kg of diets: Cu (as copper sulfate) 10 mg, Fe (as ferrous sulfate) 80 mg, Mn (as manganese sulfate) 80 mg, Zn (as zinc sulfate) 75 mg, I (as potassium iodide) 0.40 mg, Se (as sodium selenite) 0.30 mg.

^2^The vitamin premix provided per kg of diets: vitamin A, 250 000 IU; vitamin D, 50 000 IU; vitamin K3, 53 mg; vitamin B1, 40 mg; vitamin B2, 120 mg; vitamin B12, 0.50 mg; vitamin E, 600 IU; biotin, 0.65 mg; folic acid, 25 mg; pantothenic acid, 240 mg; niacin, 1 000 mg.

**Table S2** The Summary of *P*-values of genus between any two stages

| Name | *P*-value between two groups | | | | | |
| --- | --- | --- | --- | --- | --- | --- |
|  | AB | AC | AD | BC | BD | CD |
| *Alistipes* | >0.05 | 0.021 | >0.05 | 0.004 | 0.021 | >0.05 |
| *Bacteroides* | 0.013 | >0.05 | 0.002 | >0.05 | >0.05 | >0.05 |
| *Oscillospira* | 0.010 | >0.05 | 0.006 | >0.05 | <0.001 | 0.042 |
| *Bilophila* | >0.05 | <0.001 | 0.005 | 0.026 | 0.005 | 0.007 |
| *Blautia* | >0.05 | 0.006 | >0.05 | >0.05 | >0.05 | >0.05 |
| *Lactobacillus* | >0.05 | 0.031 | 0.003 | >0.05 | 0.004 | 0.005 |
| *Rikenella* | >0.05 | <0.001 | <0.001 | >0.05 | 0.001 | >0.05 |
| *Butyricimonas* | >0.05 | >0.05 | 0.006 | >0.05 | 0.005 | >0.05 |
| *Anaerofustis* | 0.031 | 0.002 | 0.003 | >0.05 | 0.020 | 0.025 |
| *Eggerthella* | >0.05 | 0.020 | >0.05 | 0.005 | >0.05 | 0.032 |

Note: A = 3 weeks; B = 6 weeks; C = 12 weeks; D = 16 weeks.

**Supplemental Figure S1**


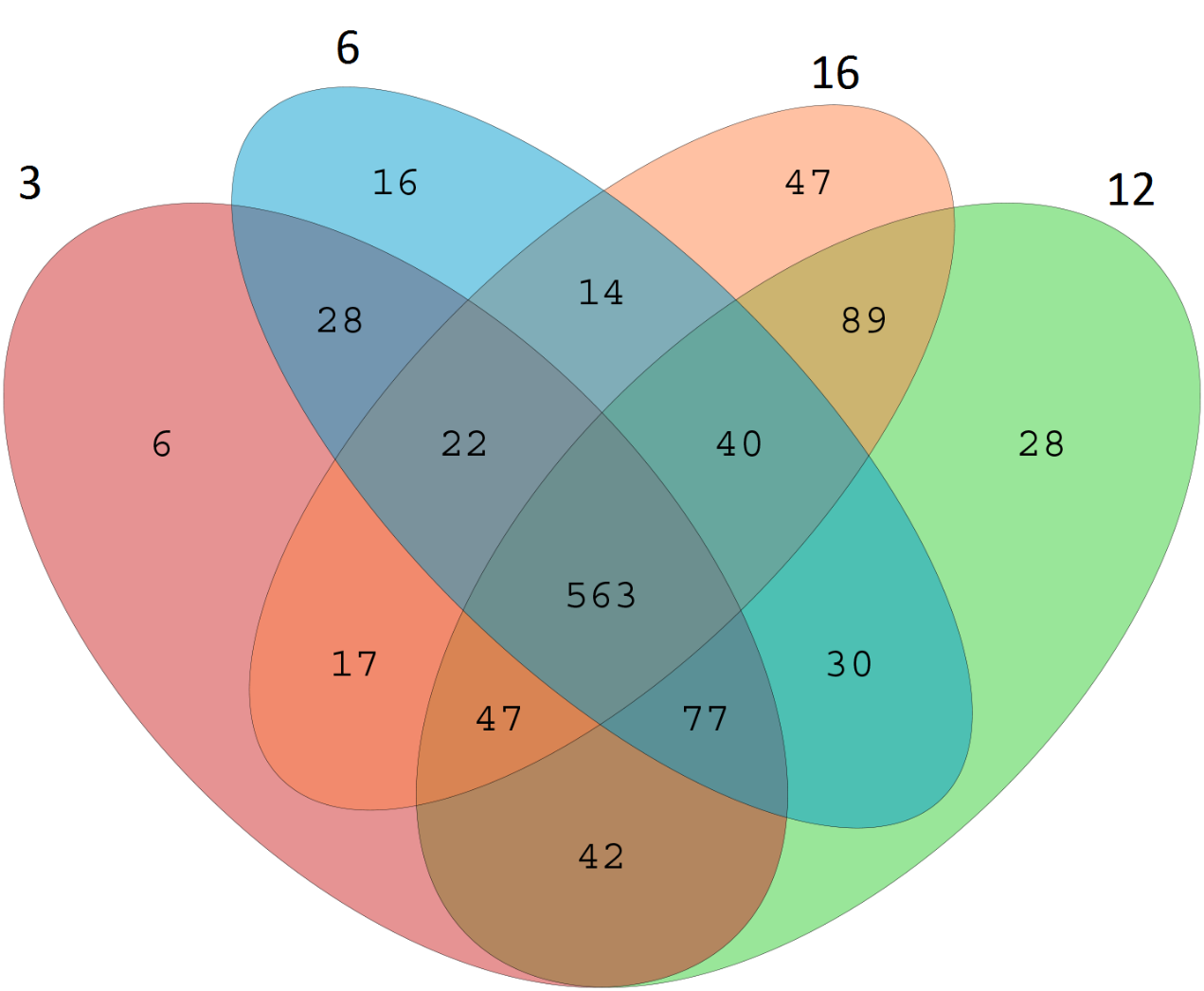


**Figure S1** The Venn diagram shows the microbes shared within different developmental stages.
