# Supplementary figures and images for: Age-associated changes in caecal microbiome and their apparent correlations with growth performances of layer pullets
Source: Anim Nutr. 2021 Jul 10;7(3):841–8. doi: 10.1016/j.aninu.2020.11.019 (PMC8379648; doi:10.1016/j.aninu.2020.11.019)

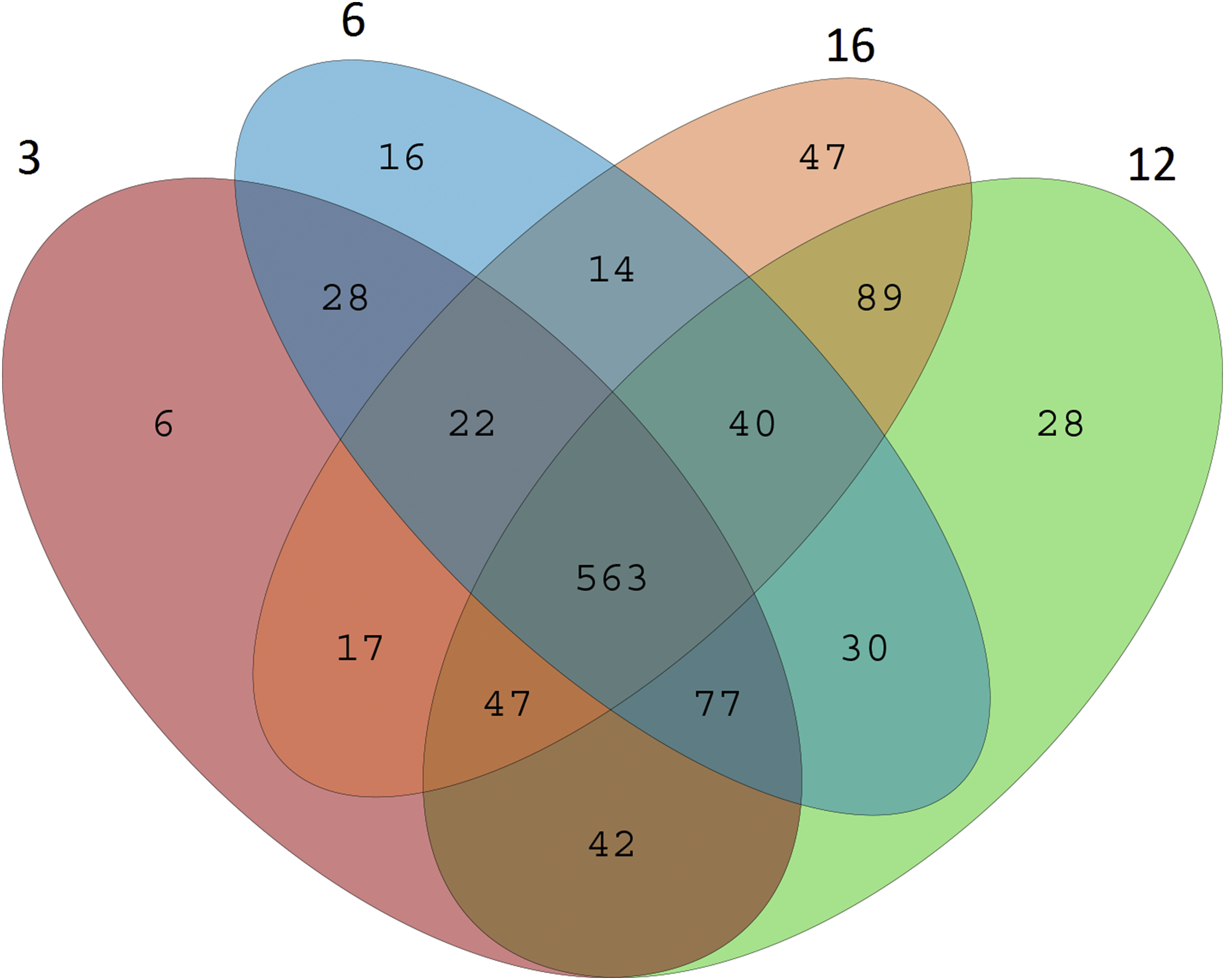

Supplement: Fig. S1 [file figs1.jpg]
